# Improved comprehension of influenza‐related headaches: Perspectives and suggestions for incidence and prevalence of headache in influenza

**DOI:** 10.1111/ene.16477

**Published:** 2024-09-12

**Authors:** Saim Mahmood Khan, Jawairya Muhammad Hussain, Areeb Bin Khalid, Ramsha Sultan

**Affiliations:** ^1^ Karachi Medical and Dental College Karachi Pakistan

Dear Editor,

Upon examination of the article “Incidence and prevalence of headache in influenza: A 2010–2021 surveillance‐based study” by García‐Azorín et al. [1], we commend the authors for their efforts to describe the occurrence and frequency of headaches in influenza patients. There are a few things to be concerned about, even if this work provides a useful investigation of headaches during influenza.

The study did not provide information regarding patients' medication use, particularly how their medications might have affected their symptoms or interacted with antiviral treatments. Furthermore, the study did not furnish detailed information concerning the patient's travel history, notably whether they had recently visited regions where influenza was prevalent. Additionally, there was no comparison of headache prevalence in influenza patients with other comorbidities such as diabetes or cardiovascular disease, which could elucidate associations with specific health conditions or comorbidities [2].

It was found that patients with perceived health in the headache state had 60% less chance of getting referred for more attention, suggesting less severe illness. Nevertheless, the investigation of the present research did not include data about those long‐term consequences that may increase mortality rate or require intensive care [3]. Furthermore, this research does not consider the re‐evaluation of the results after the participants have regained normal health, thus lacking critical information regarding the effect of current treatments on the headache experience in patients with acute infections. Furthermore, the study also did not mention any prior influenza history. It is crucial to understand the significance of patients' prior influenza history as well as the influence of headaches.

Additionally, the research fails to examine whether other psychological states such as stress and anxiety trigger a headache. The case report shows that a 16‐year‐old boy was admitted to the hospital due to influenza and COVID‐19. On the 5th day of the illness, he had hallucinations and delirium, even though he never had a drug or mental illness. It is important to note that psychotic symptoms and COVID‐19 co‐occurred in this case study [4].

Although the researchers deserve recognition for their study, which brings attention to a crucial aspect of influenza symptoms and sets the stage for further in‐depth investigations, a more impartial assessment of competing perspectives would enhance the quality of the study. Our recommendations are intended to increase the effect and reach of the writers' excellent work in the field, which already has a solid foundation.

## AUTHOR CONTRIBUTIONS

**Saim Mahmood Khan:** Writing – original draft; writing – review and editing; investigation; funding acquisition; formal analysis; software; supervision; resources; conceptualization; methodology; validation; visualization; project administration; data curation. **Jawairya Muhammad Hussain:** Methodology; formal analysis; data curation; writing – review and editing; writing – original draft; investigation. **Areeb Bin Khalid:** Investigation. **Ramsha Sultan:** Resources.

## ETHICS STATEMENT

The authors are accountable for all aspects of this work in ensuring that questions related to the accuracy or integrity of any part of the work are appropriately investigated and resolved. None of the authors serves as an unpaid editorial board member (editors‐in‐chief, editorial board member, or section editor).

## Data Availability

Research data are not shared.
